# Site and Mechanism of Recurrent Pontine Infarction: A Hospital-Based Follow-Up Study

**DOI:** 10.3390/brainsci12050520

**Published:** 2022-04-20

**Authors:** Li Wu, Youfu Li, Zeming Ye, Dezhi Liu, Zheng Dai, Juehua Zhu, Hongbing Chen, Chenghao Li, Chaowei Lie, Yongjun Jiang

**Affiliations:** 1 Department of Neurology, The Second Affiliated Hospital of Guangzhou Medical University, 250 Changgang East Road, Guangzhou 510260, China; lilywu_yeah@aliyun.com (L.W.); jarbonelee@tom.com (Y.L.); 2 Department of Neurology, Guangzhou Panyu District Hexian Memorial Hospital, 2 Qinghe East Road, Guangzhou 511400, China; yezeming2011163@163.com; 3 Department of Neurology, Shanghai General Hospital, Shanghai Jiao Tong University, 85 Wujin Road, Shanghai 200080, China; yzldz@126.com; 4 Department of Neurology, Wuxi People’s Hospital, 299 Qingyang Road, Wuxi 214023, China; lxjdz88@126.com; 5 Department of Neurology, The First Affiliated Hospital of Soochow University, 899 Pinghai Road, Suzhou 215300, China; zhujuehua@suda.edu.cn; 6 Department of Neurology and Stroke Center, The First Affiliated Hospital, Sun Yat-sen University, 58 Zhongshan Road II, Gungzhou 510080, China; shark2860@sina.com; 7 Department of Radiology, The Second Affiliated Hospital of Guangzhou Medical University, 250 Changgang East Road, Guangzhou 510260, China; lijerry13560191033@163.com (C.L.); lie393321054@gmail.com (C.L.)

**Keywords:** recurrence, pontine infarction, infarct site, branch atheromatous disease, small vessel disease

## Abstract

Although pontine infarction is the most common subtype of posterior circulation stroke, there has been little research focusing on recurrent pontine infarction. Our study aimed to investigate the factors associated with site and mechanism of recurrent pontine infarction. Patients with acute isolated pontine infarction were enrolled and followed up for one year. Lesion topography was determined by diffusion-weighted imaging. Mechanisms were determined based on lesion topography and other vascular, cardiologic and laboratory results. A total of 562 patients with pontine infarction were included, with 67 patients experiencing recurrence during the follow-up period. Forty-one recurrences occurred at the same site as index pontine infarction (41/67, 61.2%). Results indicated that the mechanism of index pontine infarction was significantly associated with the recurrent sites (*p* = 0.041, OR 2.938, 95% CI 1.044–8.268), and also with the mechanisms of recurrence (*p* = 0.004, OR 6.056, 95% CI 1.774–20.679). Branch atheromatous disease-induced index pontine infarction was likely to recur at the same site and with the same mechanism. Moreover, if recurrence occurred at the same site, the mechanism was probably the same as that of the index stroke (*p* = 0.000). Our study may help physicians treat patients with pontine infarction by predicting the site and mechanism of recurrence.

## 1. Introduction

The first and key step of preventing recurrent stroke is to understand its knowledge, especially where and why it would recur [1]. Previous studies have shown that intracranial large-artery disease would cause recurrence at the same site or adjacent to the index stroke [2], which made it possible to predict recurrence. However, the site and mechanism of recurrence caused by branch atheromatous disease (BAD) and small vessel disease (SVD) remain unclear.

Pontine infarction is the most common stroke subtype in the posterior circulation [3]. Referring to stroke etiology, BAD was the most frequent mechanism in pontine infarction (39%–44%), followed by SVD (34%) [4,5,6,7]. Large artery disease (LAD) and other mechanisms, such as potential cardiac sources of embolism (PCSE), were less common [4,5,6,7]. Pontine infarction etiology has been proven to be correlated with lesion topography [8,9]. BAD was particularly associated with large ventral infarctions reaching the pontine surface [7]. SVD was usually defined as small (<15 mm) lesions sparing the surface [3]. The prognosis of pontine infarction varies among different mechanisms. Short-term (30 days to 3 months) and long-term (9 months to 5 years) outcomes were relatively benign in patients with SVD but worse in patients with BAD [4,6,10,11,12].

Patients with BAD or SVD might have a high risk of recurrence, and some previous studies suggested a specific stroke pathophysiology in these patient subgroups [13]. In our previous publication, we conducted a multicenter study that recruited the largest cohort of patients with pontine infarction [5]. In the present study, we analyzed one-year follow-up data to elucidate the topographic locations and mechanisms of recurrent pontine infarction. To our knowledge, this study is the first to investigate the factors associated with site and mechanism of recurrent pontine stroke.

## 2. Materials and Methods

### 2.1. Study Design

**Figure 1:**
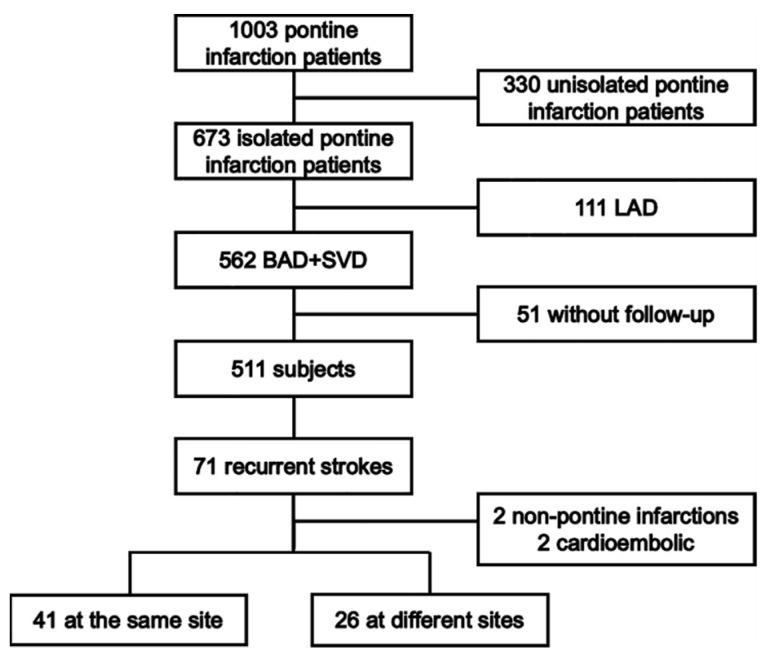
The schematic diagram of the study design.

A schematic diagram of the study design is shown in Figure 1. This was a multicenter hospital-based follow-up study that enrolled patients with index pontine infarction admitted from 1 May 2003 to 31 October 2017 (The Second Affiliated Hospital of Guangzhou Medical University, Guangzhou Panyu District Hexian Memorial Hospital, Shanghai General Hospital, Wuxi People’s Hospital, the First Affiliated Hospital of Suzhou University, and the First Affiliated Hospital of Sun Yat-sen University). The research protocol was reviewed and approved by the ethics committees of each institution. Written consent was obtained from patients or their authorized relatives before enrollment. Details were previously reported [5].

### 2.2. Study Population

Patients that met the following criteria were included: (1) over 18 years old; (2) with symptoms of stroke; (3) admitted within 7 days after onset and diagnosed with acute pontine infarction by diffusion weighted imaging (DWI); (4) with intracranial and extracranial cerebral arteries visualized by ultrasound, magnetic resonance angiography (MRA), computed tomography angiography (CTA) or digital subtraction angiography (DSA); and (5) index stroke mechanism was classified as BAD or SVD. Patients were excluded if they met any of the following criteria: (1) missing clinical or imaging information, or images could not be identified; (2) tumor, parasites, or hemangioma in the pons; (3) pontine hemorrhage or trauma; (4) a previous history of atrial fibrillation or newly diagnosed atrial fibrillation; (5) two or more mechanisms; (6) nonatherosclerotic stroke; or (7) index or recurrent stroke involving the regions outside of the pons. The patients received standard therapy including thrombolytic therapy, embolectomy or other medications at the acute stage of index stroke, and then they were given standard secondary prevention including stroke risk control, antiplatelet drugs (aspirin and clopidogrel for 3 weeks following by aspirin or clopidogrel) and standard dose of statin. All patients were followed up for 12 months (at 1, 3, 6, and 12 months after discharge) by telephone or in the outpatient department. If any suspected new neurological symptoms emerged, the patients were evaluated by one senior neurologist. If diagnosed as recurrent stroke by the senior neurologist, MRI scanning was performed to confirm the sites of new infarction at 72 h—7 d after onset.

### 2.3. Demographic Characteristics

On admission, demographic information (including age and gender), risk factors for stroke (including current smoking or drinking, hypertension, hyperlipidemia, diabetes, and previous stroke or transient ischemic attack (TIA)), and current medications on admission were acquired. Current drinking was defined as heavy intake (≥14 drinks/week in women or 21 drinks/week in men) or episodic heavy intake (≥5 drinks/episode at least once per month) [14]. Blood glucose, blood cholesterol, and cardiac examinations (electrocardiogram and echocardiogram) were also performed.

### 2.4. The Site and Mechanisms of Pontine Infarction

All patients underwent MRI using either 1.5 T or 3.0 T MRI unit. Standard axial and sagittal MRI sequences, including T1-weighted imaging, T2-weighted imaging, DWI, fluid-attenuated inversion recovery sequence, apparent diffusion coefficient maps, and time-of-flight MRA covering the circle of Willis were applied. Lesion topography was determined based on DWI with reference to other sequences. Sagittal sections were used to confirm whether there was associated infarcts in the medulla oblongata, mesencephalon and thalamus or sporadic infarct in distant areas. There were usually three axial images from rostral to caudal pons. All sections were interpreted by two independent imaging physicians who were blinded to the study design.

Based on their clinical symptoms, infarction site and size on DWI and the results of vascular, cardiologic and laboratory results, the mechanisms of index or recurrent pontine infarction could be divided into 5 categories: (1) LAD was presumed in patients with a stenosis of more than 50% of the lumen diameter in the large artery (vertebral or basilar artery) corresponding to the infarct territory, (2) BAD was presumed in patients with an infarct reaching or approaching the ventral pontine surface in the absence of LAD corresponding to the infarct and potential sources of cardio-embolism, (3) SVD was considered if the patients had a small (less than 15 mm in diameter) deep (sparing the surface of the pons) infarct lesion, in the absence of other etiologies, (4) PCSE mainly included nonvalvular atrial fibrillation, mitral stenosis, a prosthetic valve, myocardial infarction within 6 weeks, intracardiac clot, ventricular aneurysm and bacterial endocarditis, and (5) other undetermined etiologies [6].

**Figure 2:**
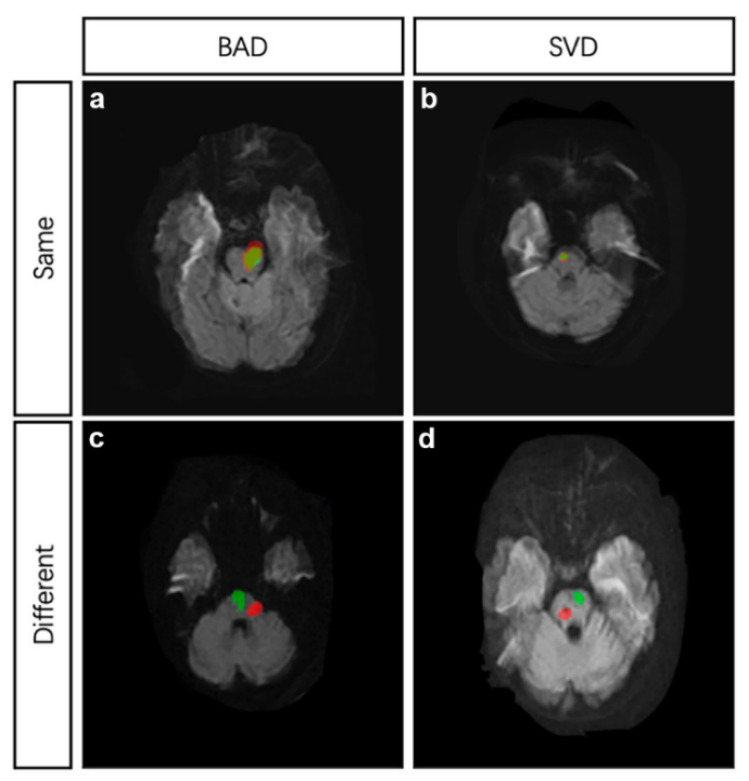
MRI demonstrations of four different conditions. (**a**), the index stroke and recurrent stroke were both attributed to BAD and located at the same site. (**b**), the index stroke and recurrent stroke were both attributed to SVD and located at the same site. (**c**), the index stroke and recurrent stroke were both attributed to BAD but located at different sites. (**d**), the index stroke and recurrent stroke were both attributed to SVD but located at different sites. Red area indicates the index infarction while green area indicates the recurrent infarction. BAD, branch atheromatous disease, SVD, small vessel disease.

To compare the site of recurrence, two DWI images in TIFF from index and recurrent stroke at the same cross section were dealt with in Photoshop 7.0. Briefly, open the first DWI image; add the secondary DWI image to another layer in the same project; adjust the transparency of the second image; and resize the second image with reference to the basilar artery and the fourth ventricle. The same site of two strokes was defined as follows (Figure 2): (1) if the index and recurrent infarctions were both BAD, and the recurrence was confirmed to be caused by the same branch artery; (2) if both the index and recurrent infarctions were SVD, and the recurrent infarction mostly overlapped with the index infarction; (3) if index infarction was SVD and recurrent infarction was BAD, and the recurrent infarction area covered the index infarction; and (4) if index infarction was BAD and recurrent infarction was SVD, only if the recurrent infarction located within the range of index infarction area would the infarct site be considered the same as index stroke. Cohen’s kappa was used to determine the intra-observer agreement. The kappa value was 0.91 (*p* < 0.05).

### 2.5. Statistical Analysis

Statistical analysis was performed using SPSS (version 20.0). Continuous variables were tested for normality using the Kolmogorov–Smirnov test. Normally distributed continuous variables are expressed as the mean ± standard deviation, while categorical variables are expressed as percentages. Differences in continuous variables were compared by Student’s t test (normally distributed) or the equivalent nonparametric test (nonnormally distributed). Univariate and multivariate logistic regression were performed to confirm the factors independently associated with the site and mechanism of recurrence. Variables with a potential association from univariate analysis (*p* < 0.2) were used for multivariate analysis. Pearson’s chi-square test was used to assess differences in the mechanisms of index and recurrent pontine infarctions. *p* < 0.05 was considered statistically significant.

## 3. Result

### 3.1. Participants

A total of 1003 patients with pontine infarction were screened, as shown in Figure 1. Of these, 330 patients with confirmed cases of unisolated pontine infarction were excluded. For the remaining 673 patients with isolated pontine infarction, 111 patients with index pontine infarction attributed to LAD were excluded. During the 12-month follow-up, 51 patients were lost. A total of 71 recurrent strokes occurred, with two located in the regions outside of the pons and two derived from cardio-embolism. The rate of recurrent pontine infarction was 13.1% (67/511). There were 21 (4.1%), 32 (6.3%), 40 (7.8%) and 67 (13.1%) stroke recurrence on 1, 3, 6, and 12 months after the index pontine infarction, respectively. There were more recurrences at the same site than at different sites (41 vs. 26). The demographic and clinical characteristics are presented in Table 1.

### 3.2. Mechanism of Index Pontine Infarction Associated with Site of Recurrence

In the univariate logistic regression analysis, antihypertensives usage and mechanisms of index pontine infarction were potentially associated with the same site of recurrence (*p* < 0.2). In the multivariate logistic regression analysis, BAD as the mechanism of index pontine infarction was significantly associated with the same site of recurrence (*p* = 0.041, OR 2.938, 95% CI 1.044–8.268, Table 1).

### 3.3. Mechanism of Index Pontine Infarction Associated with Mechanism of Recurrence

In the univariate logistic regression analysis, hypertension, diabetes, index stroke mechanism and site of recurrence were potentially associated with the mechanism of recurrence (*p* < 0.2). In the multivariate logistic regression analysis, hypertension (*p* = 0.038, OR 6.010, 95% CI 1.100–32.850, Table 2), BAD of index stroke (*p* = 0.004, OR 6.056, 95% CI 1.774–20.679, Table 2) and the same site of recurrence (*p* = 0.045, OR 3.710, 95% CI 1.030–13.364, Table 2) were significantly associated with BAD of recurrent pontine infarction. For the subgroup analysis, if recurrence of BAD-induced index stroke occurred at the same site, the mechanism of recurrence was probably BAD (*p* = 0.000, Table 3), and if recurrence of SVD occurred at the same site, it was likely to be another SVD (*p* = 0.000, Table 3). However, if recurrence occurred at different sites, the mechanism was not significantly related to the mechanism of index pontine infarction (*p* > 0.05, Table 3).

## 4. Discussion

The present study investigated the factors associated with the site and mechanism of recurrent pontine stroke. Our data showed that 13.1% of patients with pontine infarction had recurrence in the first year. The majority of recurrences occurred at the same site of the index pontine infarction. Recurrence in patients with BAD was likely to occur at the same site, while there was no such feature in patients with SVD. The mechanism of recurrence was significantly related to that of index pontine infarction. The main strength of our study was to demonstrate the relationship of the site and mechanism of index pontine infarction with the site and mechanism of recurrent pontine infarction, which would help physicians predict where and why recurrent pontine infarction is going to occur.

Pontine infarction was found to tend to recur, while few researches regarding the recurrence rate of pontine stroke has been conducted. Based on our data, the one-month, three-month, six-month and one-year recurrence rate of pontine infarction caused by BAD and SVD were 4.1%, 6.3%, 7.8% and 13.1%, respectively, which were similar to the recurrence rates of posterior circulation stroke [4,10] and higher than that of anterior circulation stroke [15,16,17]. The high rate of recurrence warrants additional research. Some factors such as concomitant presence of coronary heart disease and higher number of risk factors for atherosclerosis were found to be related to the recurrence of pontine stroke [18]. To explore the characteristics of recurrent pontine infarction, the first question was to determine where recurrent pontine infarction would happen.

In the present study, we found that 61.2% of recurrences occurred at the same site as the index stroke. Previous studies have demonstrated that large intracranial artery disease including basilar artery disease always caused recurrent stroke in the same area [1,19,20]. The recurrence pattern of large intracranial artery disease could be explained by artery-to-artery embolism [21]. For branch artery and small artery diseases, no such evidence has been reported. In this study, demographic information (including age and gender), risk factors for stroke (including current smoker or drinker, the presence of hypertension, hyperlipidemia, diabetes, and stroke or TIA history), current medications (including antihypertensives, hypoglycemics, statins, antiplatelets), and the mechanisms of index pontine infarction (including BAD and SVD) were included in univariate logistic regression analysis, then multivariate logistic regression analysis, to investigate the potential factors associated with the site and mechanism of recurrent pontine stroke. The results showed that the mechanism of index stroke was associated with the site of recurrence. Patients with BAD in the first stroke were likely to be attacked at the same site during recurrence, while there was no such feature in patients with SVD. The underlying mechanism remained undetermined. The possible reason might be that BAD was an occlusion or stenosis at the origin of one penetrating artery without affecting other penetrating arteries [3]. Thus, recurrence would be caused by thrombosis formed at the origin of the same penetrating artery. In SVD referring to random terminal artery occlusion due to lipohyalinosis or other pathogenies, there would be equal opportunity to cause recurrence at any site [22]. Thus, it was assumed that the mechanism of recurrence would be related to that of index strokes.

In the next step of the analysis, hypertension, mechanism of index stroke and recurrence at the same site were found to be significantly associated with the mechanism of recurrent pontine infarction. It is beyond our expectation that hypertension was related to the mechanism of recurrent stroke, since it is the risk factor for both branch atheromatous disease and small vessel diseases [6,7]. As expected, BAD in the first stroke was likely to cause another BAD during recurrence. In the subgroup analysis, if the recurrence occurred at the same site as the index stroke, the mechanism was probably the same as the mechanism of the index stroke. However, if recurrence occurred at different sites, the mechanism of recurrence was unpredictable.

There were some limitations to this study. First, even with the largest sampling number involving recurrence of pontine stroke, this study included a relatively small number of patients with recurrence, especially in the subgroup analysis. Then, index and recurrent pontine infarction were strictly defined according to the MRI findings. This would cause potential bias for some patients unable to receive MRI due to restrictions, such as having a pacemaker.

In conclusion, our data indicated that 13.1% of patients with pontine infarction experienced recurrence during the first year, the site and mechanism of which was associated with the index pontine infarction mechanism. Patients with BAD-induced index pontine infarction were likely to be affected at the same site during recurrence, and patients with recurrence at the same site were more likely to have the same mechanism as index pontine infarction. These phenomena might give neurologists a suggestion in predicting the site and mechanisms of recurrent pontine infarction and help them adapt appropriate prevention measures to these patients according to the infarct site and mechanism.

## Figures and Tables

**Table 1:** Site of recurrence.

	Site of Recurrence		Univariate Analysis			Multivariate Analysis		
	Same (*n* = 41)	Different (*n* = 26)	OR	*p*	95% CI	OR	*p*	95% CI
Gender (male)	28 (68.29%)	15 (57.69%)	1.579	0.379	0.570–4.375			
Age (years, m ± SD)	66.17 ± 10.59	69.35 ± 8.52	0.966	0.202	0.916–1.019			
Smoker	11 (26.83%)	7 (26.92%)	0.995	0.993	0.329–3.015			
Drinker	6 (14.63%)	3 (11.54%)	1.314	0.718	0.298–5.787			
Hyperlipidemia								
TG	17 (41.46%)	7 (26.92%)	1.923	0.230	0.662–5.584			
TC	17 (41.46%)	9 (34.62%)	1.338	0.576	0.483–3.708			
LDL	10 (24.39%)	4 (15.38%)	1.774	0.381	0.492–6.393			
Hypertension	33 (80.49%)	24 (92.31%)	0.344	0.201	0.067–1.765			
Diabetes	16 (39.02%)	13 (50.00%)	0.640	0.378	0.237–1.726			
Stroke History	12 (29.27%)	11 (42.31%)	0.564	0.276	0.202–1.578			
Medications								
Antihypertensive	19 (46.34%)	17 (65.38%)	0.457	0.131	0.166–1.261	0.493	0.186	0.173–1.405
Hypoglycemic	16 (39.02%)	10 (38.46%)	1.024	0.963	0.373–2.809			
Statins	32 (78.05%)	18 (69.23%)	1.580	0.421	0.519–4.813			
Antiplatelet	35 (85.37%)	21 (80.77%)	1.389	0.622	0.377–5.118			
Index stroke mechanism			3.086	0.030	1.112–8.559	2.938	0.041	1.044–8.268
BAD	27 (65.85%)	10 (38.46%)						
SVD	14 (34.15%)	16 (61.54%)						

TG, triglyceride, TC, total cholesterol, LDL, low-cholesterol lipoprotein, BAD, branch atheromatous disease, SVD, small vessel disease.

**Table 2:** Mechanism of recurrence.

	Mechanism of Recurrence		Univariate Analysis			Multivariate Analysis		
	BAD (*n* = 42)	SVD (*n* = 25)	OR	*p*	95% CI	OR	*p*	95% CI
Gender (male)	27 (64.28%)	16 (64.00%)	1.012	0.981	0.361–2.842			
Age (years, m ± SD)	66.47 ± 10.19	68.96 ± 9.37	0.974	0.320	0.924–1.026			
Smoker	13 (30.95%)	5 (20.00%)	1.793	0.331	0.552–5.825			
Drinker	7 (16.67%)	2 (8.00%)	2.300	0.325	0.439–12.062			
Hyperlipidemia								
TG	17 (40.48%)	7 (28.00%)	1.749	0.305	0.061–2.090			
TC	17 (40.48%)	9 (36.00%)	1.209	0.716	0.435–3.363			
LDL	8 (19.05%)	6 (2400%)	0.745	0.630	0.225–2.469			
Hypertension	38 (90.47%)	19 (76.00%)	3.000	0.119	0.755–11.922	6.010	0.038	1.100–32.850
Diabetes	21 (50.00%)	8 (32.00%)	2.125	0.154	0.755–5.984	2.297	0.193	0.657–8.030
Stroke History	14 (33.33%)	9 (36.00%)	0.889	0.824	0.315–2.511			
Medications								
Antihypertensive	22 (52.38%)	14 (56.00%)	0.864	0.774	0.320–2.338			
Hypoglycemic	18 (42.85%)	8 (32.00%)	1.594	0.379	0.564–4.505			
Statins	31 (73.81%)	19 (76.00%)	0.890	0.842	0.283–2.802			
Antiplatelet	36 (85.71%)	20 (80.00%)	1.500	0.543	0.406–5.541			
Index stroke mechanism			6.429	0.001	2.140–19.316	6.056	0.004	1.774–20.679
BAD	30 (71.43%)	7 (28.00%)						
SVD	12 (38.57%)	18 (72.00%)						
Site of recurrence			3.182	0.028	1.130–8.960	3.710	0.045	1.030–13.364
Same site	30 (71.43%)	11 (44.00%)						
Different	12 (38.57%)	14 (56.00%)						

TG, triglyceride, TC, total cholesterol, LDL, low-cholesterol lipoprotein, BAD, branch atheromatous disease, SVD, small vessel disease.

**Table 3:** Relationship of the mechanism of index pontine infarction with the site and mechanism of recurrent pontine infarction.

	Mechanism of Index Pontine Infarction		*p*
	BAD	SVD	
N	37	30	
Mechanism of recurrent pontine infarction located at the same site with index stroke			<0.05
BAD	26	4	
SVD	1	10	
Mechanism of recurrent pontine infarction located at different sites with index stroke			<0.05
BAD	4	8	
SVD	6	8	

BAD, branch atheromatous disease, SVD, small vessel disease.

## Data Availability

The data presented in this study are available on request from the corresponding author. The data are not publicly available due to ethical restrictions.
